# Infant-Associated Bifidobacterial β-Galactosidases and Their Ability to Synthesize Galacto-Oligosaccharides

**DOI:** 10.3389/fmicb.2021.662959

**Published:** 2021-05-03

**Authors:** Valentina Ambrogi, Francesca Bottacini, John O’Callaghan, Eoghan Casey, Justin van Breen, Barry Schoemaker, Linqiu Cao, Bas Kuipers, Mary O’Connell Motherway, Margriet Schoterman, Douwe van Sinderen

**Affiliations:** ^1^APC Microbiome Ireland, University College Cork, Cork, Ireland; ^2^School of Microbiology, University College Cork, Cork, Ireland; ^3^Department of Biological Sciences, Munster Technological University, Cork, Ireland; ^4^FrieslandCampina, Amersfoort, Netherlands

**Keywords:** prebiotics, gut microbiota, microbiome, bifidogenic, galacto-oligosaccharides, infant, oligosaccharides, *Bifidobacterium*

## Abstract

Galacto-oligosaccharides (GOS) represent non-digestible glycans that are commercially produced by transgalactosylation of lactose, and that are widely used as functional food ingredients in prebiotic formulations, in particular in infant nutrition. GOS consumption has been reported to enhance growth of specific bacteria in the gut, in particular bifidobacteria, thereby supporting a balanced gut microbiota. In a previous study, we assessed the hydrolytic activity and substrate specificity of seventeen predicted β-galactosidases encoded by various species and strains of infant-associated bifidobacteria. In the current study, we further characterized seven out of these seventeen bifidobacterial β-galactosidases in terms of their kinetics, enzyme stability and oligomeric state. Accordingly, we established whether these β-galactosidases are capable of synthesizing GOS via enzymatic transgalactosylation employing lactose as the feed substrate. Our findings show that the seven selected enzymes all possess such transgalactosylation activity, though they appear to differ in their efficiency by which they perform this reaction. From chromatography analysis, it seems that these enzymes generate two distinct GOS mixtures: GOS with a relatively short or long degree of polymerization profile. These findings may be the stepping stone for further studies aimed at synthesizing new GOS variants with novel and/or enhanced prebiotic activities and potential for industrial applications.

## Introduction

The human gut microbiota consists of a large number of microorganisms, some of which have shown to be positively associated with human host health and well-being (Edgar et al., 2011; Valdes et al., 2018). Among the various reported beneficial functions associated with a healthy gut microbiota are: homeostasis maintenance, protection against pathogens, harvesting nutrients and energy from our diet, and stimulation of the immune system (Lozupone et al., 2012). Changes in the human gut microbiota composition may occur at any age (Odamaki et al., 2016), and are driven by various factors, such as environment (Rothschild et al., 2018), dietary habits (De Filippo et al., 2010), delivery mode, age, use of antibiotics (Langdon et al., 2016) and occurrence of disease (Wang, 2009; Valdes et al., 2018). For obvious reasons and fueled by microbiome research, recent decades have seen a remarkable increase in scientific and public interest in associations between diet, gut microbiota and human health. A considerable amount of scientific effort has been dedicated to the development of novel strategies aimed at maintaining a balanced microbiota. Among these are the supplementation of beneficial bacteria (probiotics), and/or the administration of mostly indigestible (i.e., by the host) dietary substances (referred to as prebiotics) to specifically stimulate the proliferation and/or metabolic activity of desired bacteria in the gut (Lozupone et al., 2012; Cheng et al., 2017). It has been shown that prebiotics can be used to influence the gut microbiota composition for the benefit of host health (Hutkins et al., 2016). Based on the definition recently proposed by the International Scientific Association for Probiotics and Prebiotics (ISAPP) consensus panel in 2017 (Gibson et al., 2017): a prebiotic is “a substrate that is selectively utilized by host micro-organisms conferring a health benefit.” Galacto-oligosaccharides (GOS), together with inulin and fructo-oligosaccharides (FOS), were among the first recognized prebiotics, as they have been shown to promote growth of beneficial bacteria, in particular bifidobacteria and lactobacilli, in the human gut (Gibson and Roberfroid, 1995). Based on the ISAPP definition as mentioned above, several substrates have been exploited for their potential prebiotic activity, including inulin, inulin-type fructans or FOS and GOS (Macfarlane et al., 2008; Wilson and Whelan, 2017). Studies conducted in infants have shown that GOS increases the relative abundance of beneficial bacteria, such as bifidobacteria and lactobacilli, resulting in an intestinal microbiota composition that more closely resembles that of breast-fed infants (Fanaro et al., 2008; Giovannini et al., 2014; Sierra et al., 2015). In addition, a mixture of 90% short chain GOS (scGOS) and 10% long chain FOS (lcFOS) have shown to elicit similar effects on intestinal microbiota composition and associated metabolic profile (Boehm et al., 2002; Knol et al., 2005; Moro et al., 2005).

Primarily due to their demonstrated bifidogenicity, and supported by physicochemical stability and pleasant taste, GOS-containing products are extensively employed as functional ingredients in infant food formulations. Besides, GOS as an ingredient is also suitable for several other food applications such as beverages and bread products (Sako et al., 1999). In addition, the ability of GOS alone and as a mixture to promote particular skin conditions has attracted the interest of the cosmetic and pharmaceutical industry (Mori et al., 2016; Hong et al., 2017; Dall’Oglio et al., 2018; Suh et al., 2019). In order to render these potential applications commercially viable, reliable and large-scale GOS manufacturing technologies had to be developed. As a result, quite a substantial level of data is currently available in literature regarding GOS production (Tzortzis et al., 2005; Splechtna et al., 2006; Torres et al., 2010; Urrutia et al., 2013; Warmerdam et al., 2013; Martins et al., 2019).

Galacto-oligosaccharides are currently produced at an industrial scale using a transgalactosylation reaction catalyzed by a β-galactosidase enzyme where lactose is used as galactose donor and acceptor (Kim et al., 1997). The resulting GOS-containing product is an oligosaccharide mixture that is formed through a double-displacement reaction, which involves galactosylation and degalactosylation, and which can be enhanced by increasing the concentration of lactose (Brás et al., 2010). Among the commonly used sources of β-galactosidases (i.e., for industrial GOS synthesis) are those of fungal origin (Urrutia et al., 2013; Saqib et al., 2017). Bacterial enzymes have also been studied and employed for GOS production, including those derived from *Lactobacillus* species (Splechtna et al., 2006; Wichienchot et al., 2016), *Bacillus circulans* (Vivinal^®^ GOS) (Torres et al., 2010), *Bifidobacterium* species (Laere et al., 2000; Han et al., 2014; Viborg et al., 2014) for the production of Bimuno GOS (Goulas and Tzortzis, 2007) and *Streptococcus thermophilus* in combination with *Aspergillus oryzae* (Chen and Gänzle, 2017). β-galactosidases typically belong to the glycosyl hydrolase families GH1, GH2, GH35 or GH42, and certain members of GH2 and GH42 have been exploited for GOS synthesis (Møller et al., 2001; Goulas et al., 2007; James et al., 2016).

In a previous study we characterized the hydrolytic activity and substrate specificity of a number of β-galactosidases encoded by various infant-associated bifidobacteria (Ambrogi et al., 2019). Following this preliminary characterization, a subset of seven bifidobacterial β-galactosidases was selected in the current report for further characterization in terms of their oligomeric state, enzyme stability and kinetics, as well as their suitability for GOS synthesis.

## Materials and Methods

### Enzyme Preparation

Heterologous expression and purification of the seven bifidobacterial β-galactosidases assessed in this study (here designated as BgaA, BgaB, BgaC, BgaD, BgaE, BgaF, BgaG) (salient details of these enzymes can be found in Table 1) was carried out according to a previously described method (Ambrogi et al., 2019). Briefly, 2% of overnight cultures of *Lactococcus lactis* strains, each containing the expression plasmid pNZ8150 (Mierau and Kleerebezem, 2005) in which each of the seven His-tag-containing and β-galactosidase-encoding genes had been cloned (Ambrogi et al., 2019), were inoculated in 1.6 or 3.2 L of M17 broth (Table 2) supplemented with 0.5% glucose. Cultivation of *L. lactis* cultures was performed at 30°C until an Optical Density (OD_600__nm_) of 0.5 was reached, at which point target gene expression was induced by the addition of filter-sterilized cell free supernatant of the nisin-producing strain *L. lactis* NZ9700 (0.2% v/v) (de Ruyter et al., 1996). Following incubation at 30°C for 90 min, cells were harvested by centrifugation (8,000 × *g* for 10 min) and the obtained pellet was resuspended in lysis buffer (50 mM sodium phosphate buffer, pH 8; 300 mM NaCl; 10 mM imidazole). Cell disruption was performed by repeated bead beating (Mini BeadBeater-16, BioSpec, Bartlesville, OK, United States; three times for 1 min). Following this, debris was removed by centrifugation (10,000 × *g* for 30 min at 4°C) to produce a crude cell extract. Individual His-tagged β-galactosidases were then purified from a given crude cell extract by Fast Protein Liquid Chromatography (FPLC, Akta pure) employing a 1 ml HisTrap^TM^ Hp column (GE Health Care). Elution was performed at a constant flow rate of 1.0 ml min^–1^ using the following two buffers 100 mM Tris–HCl + 150 mM NaCl, pH7 (buffer A), and 100 mM Tris–HCl + 150 mM NaCl + 250 mM Imidazole, pH7 (buffer B). The seven β-galactosidases were individually purified employing a standard linear elution gradient. The purity of the obtained enzymes was analyzed by SDS-polyacrylamide gel electrophoresis (SDS-PAGE), as described previously (Laemmli, 1970), on a 12.5% polyacrylamide (PAA) gel. SDS-PAGE gels were fixed and stained with Coomassie brilliant blue to identify fractions containing the purified protein and to assess purity. The elution fractions containing a given purified protein were selected, pooled and dialyzed against 40 mM sodium citrate buffer at pH 6.5 employing Centrifugal Filter Units with a 30 kDa cut-off (Merck Millipore Ltd.). Protein concentrations were determined by the Coomassie Brilliant blue method with the use of bovine serum albumin to generate a standard calibration curve (Ernst and Zor, 2010).

**TABLE 1:** Description of the enzymes employed.

**Locus tag**	**Origin**	**GH family**	**Name**	**Accession number**
Bbr_0010	*B. breve* UCC2003	GH2	BgaA	ABE94727.1
Bbr_0529	*B. breve* UCC2003	GH42	BgaB	ABE95226.1
B216_08266	*B. bifidum* LMG 13195	GH42	BgaC	EKE50024.1
B8809_0415	*B. longum* subsp. *longum* NCIMB 8809	GH42	BgaD	ALO72088.1
B8809_0611	*B. longum* subsp. *longum* NCIMB 8809	GH2	BgaE	ALO72284.1
B8809_1361	*B. longum* subsp. *longum* NCIMB 8809	GH2	BgaF	ALO73032.1
Blon_2016	*B. longum* subsp. *infantis* ATCC 15697	GH42	BgaG	ACJ53083.1

**TABLE 2:** β-galactosidase purification characteristics.

**Enzyme**	**Starting volume (L)**	**Concentration after Dialysis (mg/ml)**	**Volume after Dialysis (ml)**	**Enzyme yield (mg)**	**Approximate Purity (%)**
BgaA	3.2	1.8	4	7.2	>95
BgaB	1.6	3.3	5	16.5	>95
BgaC	1.6	1.95	5	9.75	>95
BgaD	1.6	2.03	4	8.12	>95
BgaE	1.6	3.71	3	11.13	∼80
BgaF	3.2	1.6	4	6.4	∼80
BgaG	3.2	1.55	3	4.65	>95

### Absolute Mass Determination of β-Galactosidase Enzymes

Molecular weights of denatured protein monomers were estimated by SDS-PAGE and comparison to a Prestained Protein Marker reference (Broad Range (7–175 kDa); New England BioLabs, Hertfordshire, United Kingdom). These estimated molecular weights were compared to the calculated mass values based on the corresponding gene sequence (including the His-tag-encoding sequence) employing the ExPASy Bioinformatics Resource Portal (SIB Swiss Institute of Bioinformatics). In order to determine the absolute mass of the native form the enzymes, size exclusion chromatography was first carried out on an AKTA Pure HPLC system (GE Healthcare, Cork, Ireland) using a Superose 6 10/300 G/L column (GE Healthcare, Cork, Ireland) run in a buffer containing 50 mM Tris–HCl and 150 mM NaCl at pH 7.5 with a flow rate of 0.5 mL/min. Proteins were injected at a final concentration as listed in Supplementary Table 1. Detection was performed using OmniSec REVEAL, a dual-angle light-scattering apparatus and refractometer (RALS/LALS/RI) (Malvern Instruments, Malvern, United Kingdom). Absolute mass calculations were performed employing the OmniSec software (v10.4).

### Assessment of β-Galactosidase Activity

β-galactosidase activity of the purified enzymes was quantified by using *o*-nitrophenyl-β-D-galactopyranoside (ONPG) or lactose as substrates. The ONPG-based assay was carried out at 40°C in 40 mM sodium acetate buffer (pH 6.5) as follows: 880 μl of citrate buffer was pre-heated for 5 min at 40°C, after which 20 μl of citrate buffer containing the purified enzyme was added (representing 10 μg of purified protein in the case of BgaA, BgaE, BgaF, and BgaG; 2.5 μg in the case of purified BgaB and BgaD; 1 μg in the case of purified BgaC). Then, 100 μl of substrate solution containing ONPG at each of the following concentrations was added: 0.83, 1.66, 3.32, 6.64, 9.96, 13.28, 33.20, and 66.39 mM. After 30 s of incubation at 40°C the reaction was terminated by the addition of 200 μl of 1 M Na_2_CO_3_. The release of *o*-nitrophenol (*o*NP) was measured spectrophotometrically at 420 nm.

When lactose was employed as a substrate, the enzymatic activity of the purified β-galactosidases was determined quantitatively using the D-glucose oxidase/peroxidase GOPOD assay kit (Megazyme, Bray, Ireland) according to a previously published protocol (Benjamins et al., 2014). For this assay, 5 ml of 12% lactose solution was pre-heated into a water bath at 40°C for 10 min, after which 1 ml of sample solution was added to the reaction tube. Following 10 min incubation, the reaction was stopped by the addition of 1 ml of 1.5 M sodium hydroxide. The reaction mixture was cooled in ice water and 1 ml 1.5 M of HCl was added. The release of D-glucose from lactose was determined using the GOPOD method (Megazyme). One lactase unit (LU) was defined as the amount of enzyme that releases 1 μmol of D-glucose per minute (at the non-limiting lactose concentration used in this assay) at 40°C and pH 6.0, and individual enzyme activities were calculated employing the formula indicated below:

$$L\,u/g=\frac{G\,t-G\,b}{0.18}\times 8\times \frac{1}{10}\times \frac{1}{W}=\frac{\left(G\,t-G\,b\right)}{0.225\times W}$$

Gt = Glucose concentration of the sample solution (mg/ml)

Gb = Glucose concentration of the blank (mg/ml)

0.18 = Amount of glucose, in mg, equivalent to 1 μmol

8 = Total volume of the reaction mixture (in ml)

10 = Reaction time of 10 min

W = Weight in grams of the enzyme in the sample solution

Enzyme stability over time at different storage conditions was evaluated by employing LU determination as follows. Enzymes were stored −20°C following purification and subsequent dialysis in the dialysis buffer [40 mM sodium citrate (Na_3_C_6_H_5_O_7_)] pH 6.5 with or without the addition of 20% glycerol. LU determination was performed at various time points: immediately after enzyme purification and dialysis, after one and 2 weeks, as well as after one, two and three months. In the case of proteins stored in dialysis buffer with glycerol, an additional time point at 4 months was also assessed. Statistical analysis was performed using one-way ANOVA, followed by Tukey’s *post hoc* test. A *p*-value of less than 0.05 was considered significant.

### Optimal Conditions for Lactose Hydrolysis

Optimal lactose hydrolysis conditions of the seven purified β-galactosidases were determined for each enzyme by first implementing the above described GOPOD method. Enzyme reactions were then terminated at various time points (*t*) set at 1, 3, and 10 min. In order to determine the optimum temperature for lactose hydrolysis, the assay was conducted at various temperatures (35, 40, 45, 50, 55, and 60°C). The assay was then performed at the determined optimal temperature at pH 5 and 6 in 0.1 M sodium acetate buffer (C_2_H_3_NaO_2_) as well as pH 6, 7, and 8 in 0.1 M sodium phosphate buffer in order to assess the pH optimum of the enzyme reaction. Enzyme activity was expressed as LU/g for each temperature and pH condition using the formula indicated in the previous paragraph.

### Enzyme Kinetics

Steady-state kinetic measurements were obtained using ONPG as a substrate, while enzyme reaction conditions were set at 40°C in 0.1 M sodium acetate buffer (pH 6.5) and with substrate concentrations ranging between 0.83 and 66.39 mM. One unit of β-galactosidase activity refers to the amount of enzyme required to release 1 μmol *o*NP (from the ONPG substrate) per minute at the applied temperature and pH conditions. The catalytic properties of the seven purified enzymes were determined according to the Michaelis-Menten kinetics model, where the maximum enzyme velocity (V_max_) is extrapolated from the equation that calculates enzyme activity (Y) as a function of substrate concentration (X). The corresponding Km, which is calculated based on the concentration that causes V_max_ to halve, was determined according to the following equation:

$$Y=\mathrm{Et}*\text{kcat}*X/\left(K_{\text{m}}+X\right)$$

Et represents the number of active sites present in the enzyme and kcat is the rate at which enzymes can convert substrate to product. All parameters were determined using Graph Pad Prism version 5 (Graphpad Software, United States).

### Galacto-Oligosaccharide (GOS) Synthesis

Galacto-oligosaccharides synthesis assay was performed following a previously described method (Benjamins et al., 2014). The reaction was initiated following the addition of purified bifidobacterial β-galactosidase (to which water had been added to bring it to a total volume of 2 ml) to a lactose substrate slurry, consisting of 7.5 g lactose monohydrate (99% pure, Lactochem^®^ Super Fine Powder, DMV-Fonterra Excipients GmbH & Co., Goch, Germany), corresponding to a final concentration of 48%, 5.1 g H_2_O, 150 μl of 1 M citrate buffer (pH 7.0) and 75 μl of 1 M MgCl_2_. The citrate buffer was employed to prevent acidification of the reaction and interference with enzyme activity for the duration of the experiment.

The enzyme dose used to initiate a given GOS reaction varied between 2.5 and 8 LU per gram of lactose, depending on the enzyme. The GOS synthesis reaction was performed at 50°C in a vessel (Wide neck clear GL50, VWR) under constant stirring for a period of 32–54 h.

### Preliminary Compositional Analysis of GOS Preparations as Based on Glyco-Profiling by HPAEC-PAD

Preliminary compositional analysis of each obtained GOS mixture was determined by glyco-profile analysis employing a High Performance Anion Exchange Chromatography and pulsed amperometric detection (HPAEC-PAD; Dionex IC-3000 system; Thermo Scientific^1^). Separations were performed using a CarboPac PA1 (Thermo Scientific) analytical-anion exchange column (dimensions, 250 mm by 4 mm) with a CarboPac PA1 (Thermo Scientific) guard column (dimensions, 50 mm by 4 mm) and a detector (ED40) in the pulsed amperometric detection PAD mode (Dionex, Thermo Scientific). Qualitative analysis of the GOS Dionex profile was performed with an elution gradient according to a previously published method (Van Leeuwen et al., 2016) and the qualitative determination of the carbohydrate composition was performed by the use an elution gradient summarized in supplemental Supplementary Table 3 at a constant flow rate of 1.0 ml min^–1^ at 30°C using the following eluents with programmed gradient for the analysis: (A) 100 mmol NaOH, (B) 100 mmol NaOH, 500 mmol sodium acetate (NaAC), (C) 50 mmol NaAC and (D) Milli-Q water. The obtained chromatography profiles were analyzed employing CHROMELEON software Version 7 (Dionex, Thermo Scientific).

## Results and Discussion

### Enzyme Preparation

The seven enzymes of interest were selected from a set of seventeen previously assessed GH2 and GH42 β-galactosidases encoded by various infant-derived bifidobacteria (Ambrogi et al., 2019). These seven enzymes (here named BgaA, BgaB, BgaC, BgaD, BgaE, BgaF, and BgaG; for salient features see Table 1) were selected based on their relatively broad substrate range and high catalytic activity toward lactose (Ambrogi et al., 2019).

Heterologous expression and purification of the seven selected proteins were performed according to a method reported in a previous study (Ambrogi et al., 2019). Of note, the purification protocol for these seven His6-tagged proteins was increased in scale using a starting volume of 3.2 L of enzyme-overexpressing bacterial culture (see section “Materials and Methods”). The final protein yields varied depending on the particular β-galactosidase purified: BgaG yield was the lowest at 4.65 mg, while BgaB produced the highest protein yield at 16.5 mg (Table 2). In all cases, the purification generated sufficient amounts of purified enzyme to perform further characterization and evaluation of transgalactosylase activity of these β-galactosidases. The purity of the obtained purified proteins was visually estimated to be higher than 95% for BgaA, BgaB, BgaC, BbgaD, and BgaG, while being around 80% for BgaE and BgaF due to some minor, non-specific protein bands visible in the corresponding SDS-PAGE gels (Supplementary Figure 1).

### Optimization of Hydrolysis Conditions

**FIGURE 1:**
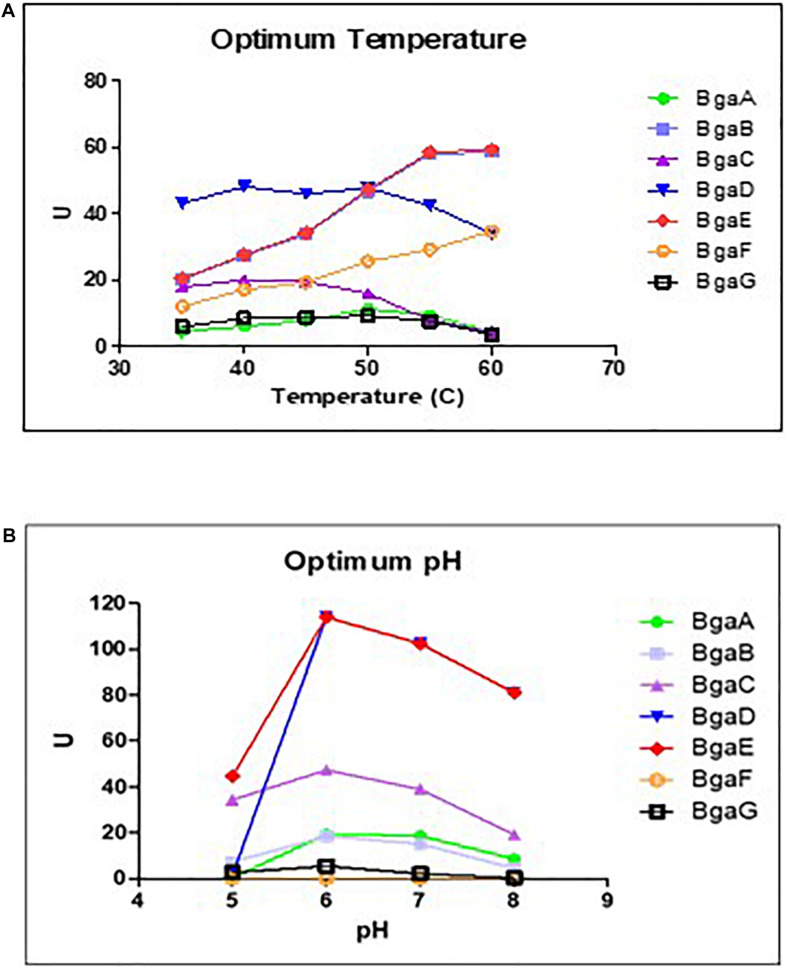
Effects of pH and temperature on enzyme activity. Determination of optimum temperature **(A)** and the optimum of pH **(B)** on enzyme activity of the seven bifidobacterial β- galactosidases Data represent the rate extrapolated by plotting enzyme activity obtained from three independent experiments (mean). The experiments conducted to investigate the optimum temperature were performed at pH 6, while the experiments aimed to determined the optimum pH were performed at the best temperature obtained.

In our previous study a qualitative assay of the seven selected enzymes (BgaA-G) established that these β-galactosidases were shown to exhibit variable substrate specificity, although they are all capable of hydrolyzing lactose (Ambrogi et al., 2019). In order to establish optimal lactose hydrolysis conditions the preferred temperature and pH for each of these seven enzymes were determined. These assays, which were conducted at a pH ranging between 6 and 8 and at various temperatures (between 35 and 60°C), showed that the seven purified β-galactosidases elicit optimal lactose hydrolysis activity at neutral conditions and a temperature range between 40 and 60°C (Figure 1; Supplementary Table 3), which is comparable to other described β-galactosidases (Osman et al., 2014). These results then served to assess the kinetic parameters of these enzymes as described in the paragraph below (which were conducted at a temperature of 40°C and pH 6.5).

### Enzyme Characterization

**FIGURE 2:**
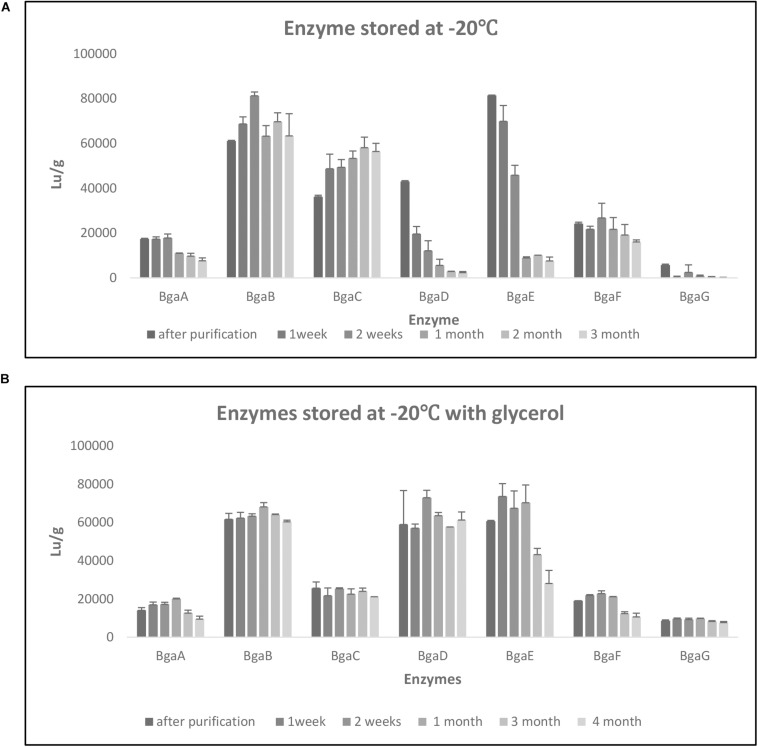
Enzyme stability. Evaluation of enzyme stability under different storage conditions at –20°C for 3 months **(A)** and –20°C with glycerol for 4 months **(B)**. Data represent the mean of two independent experiments. The enzymatic activity was express in Lu/g as per Materials and Methods.

In order to perform a kinetic assessment of the seven selected β-galactosidases, their hydrolytic activities toward ONPG and lactose were investigated. Assays for either substrate were conducted at 40°C in 0.1 M sodium acetate buffer (pH 6.5). As expected and in accordance to our previous findings (Ambrogi et al., 2019), all enzymes were shown to hydrolyze both ONPG and lactose. In the case of ONPG as a substrate, the steady-state kinetic constants were determined (V_max_, K_m_, K_cat_, and K_cat_/K_m_) and the K_cat_ values were calculated based on V_max_ values obtained from non-linear regression (assuming the presence of a single active site) (Table 3). The K_m_ values were shown to be quite variable across the enzymes tested and ranged between 114.9 mM for BgaG (highest) and 5.178 mM for BgaF (lowest), thereby indicating that BgaG has the lowest affinity for ONPG. The enzyme velocity and the consequent catalytic efficiency (K_cat_/K_m_) values were highest for BgaB and BgaC under the conditions tested. The catalytic efficiency obtained was in line with a previous study, where the BbgII enzyme (which is a homolog of BgaB) was shown to exhibit a similar Kcat/Km value under comparable test conditions (Goulas et al., 2009). Of note, enzymes BgaB and BgaC represent β-galactosidases, homologs of which are widespread across infant-derived bifidobacteria (Ambrogi et al., 2019). In particular, BgaB (product of the gene Bbr_0529 in *B. breve* UCC2003) was previously described as required for the utilization of GOS and certain HMOs (O’Connell Motherway et al., 2013; James et al., 2016; Ambrogi et al., 2019). Of note, under the conditions tested, BgaG resulted in being the least efficient enzyme among the seven assessed β-galactosidases, while also exhibiting the lowest lactase activity (LU/g; Supplementary Table 3). Its homolog β-galIII in *B. longum* subsp. *infantis* HL96 (the two enzymes share 100% amino acid sequence identity) has previously been reported to possess a rather low lactose-associated hydrolytic rate (Hung et al., 2001), in agreement with our observation of low lactase activity (Supplementary Table 3). In order to investigate the stability of the seven enzymes when kept at a low temperature, changes in lactase activity of individual enzymes over time (up to 4 months) were assessed during enzyme storage at −20°C with or without the addition of 20% glycerol. Storage of the seven enzymes at −20°C resulted in a significant decrease activity after 1 month for BgaA and BgaE, while in the case of BgaD activity reduction was already clearly observed after 1 week storage at −20°C (Supplementary Figure 2). Conversely, BgaC activity appeared to gradually (and significantly) increase during the 3 months of testing, a phenomenon for which we do not have any plausible explanation. In contrast, BgaG lost all activity within 1 week (Figure 2, panel a, Supplementary Figure 2A). BgaB showed a significant difference only between the activity measured after purification and the activity at time point 2 weeks (Figure 2, panel a; Supplementary Figure 2A). In the case of BgaF no significant activity difference was observed between the first measurement and all subsequent time points assessed (Figure 2, panel a; Supplementary Figure 2A). Addition of 20% *v*/*v* glycerol to the enzyme preparations was shown to markedly enhance the stability of enzyme activity of some of the seven β-galactosidases (Figure 2, panel b). In particular, enzyme BgaG was shown to retain activity for the entire duration of the assay with no significant difference between the first measurement and all time points considered during the assay. BgaB, BgaC, and BgaD did not suffer from any significant activity decrease (Figure 2, panel b; Supplementary Figure 2B). Surprisingly, addition of glycerol for some unknown reason appeared to reduce BgaC activity, which then remained stable upon storage. For enzymes BgaA, BgaE and BgaF activity reduction was observed following one and 3 months of storage (Figure 2, panel b; Supplementary Figure 2B). In conclusion, the obtained results show that the addition of 20% *v*/*v* glycerol to the enzyme preparations substantially improves stability of at least some of the assessed enzymes.

**TABLE 3:** Kinetic characterization of enzymes using ONPG as a substrate.

**Enzyme**	**V_max_**	**K_m_**	**K_cat_**	**K_cat_/K_m_**
**BgaA**	105.7	10.8	1,227,000	1.14E + 05
**BgaB**	586.8	8.607	18,070,000	2.10E + 06
**BgaC**	512.4	38.26	39,960,000	1.04E + 06
**BgaD**	539	17	16,600,000	9.76E + 05
**BgaE**	42.46	11.12	484,058	4.35E + 04
**BgaF**	50.03	5.178	590,354	1.14E + 05
**BgaG**	177	114.9	1,310,000	1.14E + 04

### Molecular Mass Determination

The seven investigated bifidobacterial β-galactosidases are all members of GH2 or GH42^2^ families, and exhibit a rather broad substrate specificity (Ambrogi et al., 2019). In order to obtain a better understanding of the structural properties and oligomeric state of the seven bifidobacterial β-galactosidases, the absolute molecular mass of the native enzymes was experimentally determined by size exclusion chromatography coupled to a dual-angle light-scattering apparatus (see section “Materials and Methods”). Comparison between the predicted protein monomer sizes and the experimentally obtained molecular masses of the native enzymes showed that the native form of BgaA is a dimer, while those of BgaB, BgaC, BgaD, and BgaG appear to assemble as trimmers, and that the native state of BgaE is a tetramer (Supplementary Table 1).

Notably, BgaB, BgaC, and BgaG are proteins with sequence similarity above 70%, while it has previously been reported that Bga42A (which represents a homolog of BgaG) is also active as a trimer (Yoshida et al., 2012), being consistent with our observations (Supplementary Table 1). Also in agreement with our data is that β-gal I from *B. breve* DSM 20213, which is a homolog of BgaA (99% protein sequence similarity) has been reported to form an enzymatically active dimer (Arreola et al., 2014). In contrast, Bga2A of *B. longum* subsp. *infantis* ATCC15697 (a homolog of BgaE with 99% sequence similarity) has been reported to form an enzymatically active dimer (Yoshida et al., 2012). In conclusion, our analysis shows that the seven bifidobacterial β-galactosidases represent GH2 and GH42 family enzymes that are active in various oligomeric states.

### GOS Synthesis

Transgalactosylation activity was assessed in order to determine if the seven bifidobacterial β-galactosidases are capable of GOS synthesis using lactose as the galactose acceptor and donor. Transgalactosylation occurs when β-galactosidase, following lactose hydrolysis, transfers the released galactose to another lactose unit as acceptor (instead of the hydroxyl group of water), thus resulting in the formation of oligosaccharides with a higher degree of polymerization (Kim et al., 1997). A GOS synthesis assay was performed at a temperature of 50°C with an starting lactose level of 50% (w/v) and employing an initial enzyme concentration of 4 LU per gram of lactose. Furthermore, enzyme levels corresponding to 2 LU or 4 LU per gram of lactose were added, respectively, after 15 and 22 h from the beginning of the reaction. The reactions were run over a period of 54 h. Based on the obtained results the enzymes BgaA, BgaD, BgaE, and BgaF were able to clarify the lactose slurry within the duration of the experiment. In contrast, BgaB, BgaC, and BgaG were shown to be unable to completely clarify the lactose slurry under the conditions tested, indicating that compared to BgaA, BgaD, BgaE, and BgaF, the BgaB, BgaC, and BgaG enzymes were apparently less efficient in lactose hydrolysis under the conditions used.

**FIGURE 3:**
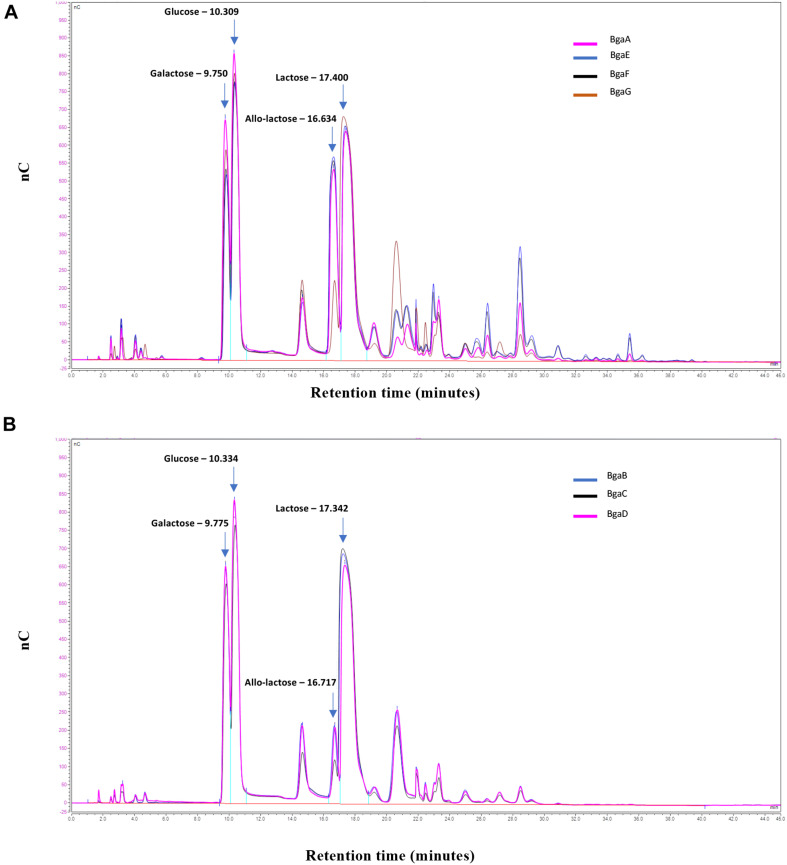
GOS synthesis. HPAEC/PAD elution patterns of the oligosaccharides obtained by transgalactosylation activity of BgaE, BgaE, BgaF or BgaG **(A)** and BgaB, BgaC or BgaD **(B)** nC: nanoCoulomb (Quantitative currency measure proportional to the carbohydrate level).

Samples of the transglycosylation reaction were taken at the end of the experiment, and the carbohydrate contents of the obtained reaction mixtures were evaluated by HPAEC-PAD (see section “Materials and Methods”). The generated chromatograms revealed that all seven enzymes are capable of transgalactosylation and that the reactions produce a mix of different mono- and oligosaccharides (Figure 3). Based on available carbohydrate standards, we identified galactose, glucose, allo-lactose and lactose (corresponding to peaks with retention times of 9.7, 10.3, 16.6, and 17.4 min, respectively), while also revealing a range of additional peaks that are presumed to represent various GOS with apparent different chain lengths and/or glycosidic linkages (Figure 3).

It is worth noting that the transgalactosylation reactions involving BgaA, BgaE, BgaF, and BgaG produced what we identified as a GOS mixture with a relative long retention profile (here named group A), including peaks with a retention time of up to 40 min (Figure 3, panel a), possibly representing oligosaccharides with a relatively high degree of polymerization (DP). In contrast, BgaB, BgaC, and BgaD produced a GOS mixture with a relative short retention profile (designated here as group B) (Figure 3, panel b), possibly representing oligosaccharides with a relatively low DP. HPAEC quantitative analysis of the reaction mixtures indicated further differences between the seven enzymes with regards to their transgalactosylase activity. The obtained results confirmed that all seven enzymes tested were able to hydrolyze lactose, furthermore they were all shown to be capable of intra- (direct galactosyl transfer to D-glucose yields regioisomers of lactose) and inter- (transfer of galactose to acceptors other than water) molecular transgalactosylation, though at apparently varying efficiencies. For example, the highest efficiency of GOS production was obtained with BgaE with final GOS (including allo-lactose) levels of 54.0%, which was also associated with the lowest remaining lactose content at 16.6% (Table 4). A less efficient GOS production level was observed for BgaC and BgaG, with, respectively, 13.74 and 14.22% of final GOS (including allo-lactose), combined with higher levels of remaining lactose, respectively, corresponding to 50.52 and 70.81% (Table 4). The examples mentioned above suggest that, under the conditions used, BgaE is an enzyme suitable for lactose conversion into GOS. In contrast, under the same conditions, BgaG was not able to effectively convert lactose in to GOS through transgalactosylation, or even into glucose and galactose through hydrolysis. A reason for this difference may be that galactose acts as an inhibitor for the enzymatic reaction, as reported previously for several β-galactosidases (Jørgensen et al., 2001; Albayrak and Yang, 2002; Torres and Batista-Viera, 2012). Interestingly, enzymes belonging to group B and producing a short elution profile, are also members of the GH42 family, while enzymes from group A (with the exception of BgaG) that generate a longer elution profile are members of the GH2 family (Table 1). It is known that the structural conformation of the active site of a given β-galactosidase impacts on the transgalactosylation/hydrolysis ratio, thereby resulting in the production of chemically different GOS mixtures (Juers et al., 2012), being consistent with our observations. In addition, GH2 family enzymes are reported to utilize lactose as their primary or natural substrate (Husain, 2010; Rodriguez-Colinas et al., 2013), which is in line with our previous observations for BgaA, BgaE, and BgaF (which are all GH family two members) (Ambrogi et al., 2019) and with the observation that they represent the most active enzymes in our GOS synthesis assay (i.e., capable of clarifying the lactose slurry within the first 8 h from the start of the reaction). In contrast, members of GH42 family are more active toward various non-lactose substrates containing β-linked galactose moieties (Husain, 2010; Rodriguez-Colinas et al., 2013). Indeed, BgaB, BgaC, BgaD, and BgaG were previously reported to be highly active toward galactobioses (Galβ1-6Gal and Galβ1-4Gal) and β-D-galactotriose (Galβ1-4Galβ1-4Gal) (Ambrogi et al., 2019), where they also represent enzymes that appear to be less efficient in our GOS synthesis attempts.

**TABLE 4:** HPAEC quantitative analysis of obtained GOS mixtures.

	**Galactose (%)**	**Glucose (%)**	**Allo-lactose (%)**	**Lactose (%)**	**Lactulose (%)**	**GOS (%)**	**GOS + Allo-lactose (%)**
**BgaA**	11.7	18.2	8.0	50.9	1.5	9.7	17.7
**BgaB**	17.9	25.0	1.8	38.0	1.2	16.2	18.0
**BgaC**	14.9	19.5	0.8	50.5	1.3	12.9	13.7
**BgaD**	19.1	26.9	3.3	31.0	0.8	18.9	22.2
**BgaE**	8.2	20.6	18.8	16.6	0.6	35.2	54.0
**BgaF**	15.5	29.1	15.1	18.9	0.8	20.5	35.6
**BgaG**	4.8	8.4	4.3	70.8	1.9	9.9	14.2

Taken together, our findings clearly show that all seven tested bifidobacterial β-galactosidases are capable of producing GOS, but they differ significantly in terms of lactose-to-GOS conversion efficiency (resulting in high GOS and low lactose content in the final reaction product). From the preliminary chromatography analysis of produced GOS it seems that two distinct GOS mixtures are generated: GOS mixtures with short and long profiles. Further optimization of transgalactosylation conditions for each of the enzymes will be necessary in order to increase GOS content. In addition, further assessment will need to be performed in order to characterize the obtained GOS structures in more details, and to establish whether the GOS mixtures possess beneficial functions. Ultimately, this will allow selection of the most promising candidate(s) for future applications.

## Conclusion

In the current study, seven β-galactosidases originating from infant-derived bifidobacteria were heterologously expressed and characterized in terms of their kinetics, storage stability, oligomeric state and suitability for GOS synthesis. Our analyses show that BgaG obtained from *B. longum* subsp. *infantis* is the enzyme with the lowest affinity for ONPG and lowest lactase activity, while BgaB and BgaC possess the highest velocity and catalytic efficiency among the seven β-galactosidases tested using ONPG as a substrate. Evaluation of enzyme stability during cold storage showed that the addition of glycerol allowed a substantially longer storage time without significantly affecting hydrolytic activity. Furthermore, molecular mass determination by size exclusion chromatography established that the seven selected enzymes assume different oligomeric conformations in solution and assemble in either dimers, trimmers or tetramers, thus confirming the heterogeneity in bifidobacterial β-galactosidases.

Finally, all characterized enzymes were shown to possess transgalactosylation activity and are to varying extents capable of synthesizing GOS mixtures, some of which appear to be of distinct composition, although this will require further characterization. Based on our findings, it appears that BgaE represents the most efficient enzyme for GOS synthesis, at least under the conditions tested, thereby making this the most promising candidate from a GOS production perspective. Future research is needed to further characterize the generated GOS mixtures and to explore their ability to elicit functional benefits. Overall, this work highlights the potential of infant-derived bifidobacterial β-galactosidases to be exploited for the development of dietary GOS.

## Data Availability Statement

The original contributions presented in the study are included in the article/Supplementary Material, further inquiries can be directed to the corresponding author.

## Author Contributions

MS and DS conceived the study. VA, JO’C, MO’C, LC, and BK designed the experiments. VA, JB, EC, and BS carried out the experiments. VA and BS analyzed the data. VA, FB, JO’C, DS, MS, LC, BK, and BS wrote the manuscript. All authors discussed the results and commented on the manuscript.

## Conflict of Interest

MS, BK, and LC are employees of FrieslandCampina. The remaining authors declare that the research was conducted in the absence of any commercial or financial relationships that could be construed as a potential conflict of interest.

## Footnotes

1 https://www.thermofisher.com/

2 http://www.cazy.org/
