# Supplementary material for: Infant-Associated Bifidobacterial β-Galactosidases and Their Ability to Synthesize Galacto-Oligosaccharides
Source: Front Microbiol. 2021 May 3;12:662959. doi: 10.3389/fmicb.2021.662959 (PMC8126724; doi:10.3389/fmicb.2021.662959)
Supplement: Supplementary file 1 [file Data_Sheet_1.pdf]

SUPPLEMENTARY MATERIAL

Table S1. Molecular mass determination and enzyme oligomerization.

Enzyme	Concentration (mg/ml)	Volume (ml)	Predicted size (kDa)	Predicted size + His-tag (kDa)	Absolute MW	Deduced oligomerization state
BgaA	1.1	3	116	117	260.9-	Dimer
BgaB	2.92	5	77	78	236.5	Trimer
BgaC	3.15	7	78	79	236.4	Trimer
BgaD	3.29	5	77	78	238.7	Trimer
BgaE	1.97	3	110.75	111.75	404	Tetramer
BgaF	2.41	3	122	123	322	Trimer
BgaG	0.91	3	74	75	246.9	Trimer

Table S2. Quantification elution method HPAEC-PAD.

Time (min)	Eluent (%)			
	A	B	C	D
0	5	0	5	90
5	5	0	5	90
20	17.5	0	50	32.5
25	17.5	30	50	32.5
25.1	0	100	0	0
30	0	100	0	0
30.1	5	0	5	90
45	5	0	5	90

Table S3. Optimal conditions where each enzyme exhibited the highest activity.

Enzyme	LU/g	Temperature (°C)	pH
BgaA	17,064.05	50	6
BgaB	60,776.06	60	6
BgaC	35,218.95	40	6
BgaD	42,465.33	40	6
BgaE	67,554.93	55	6
BgaF	25,167.52	60	6
BgaG	6,311.36	50	6

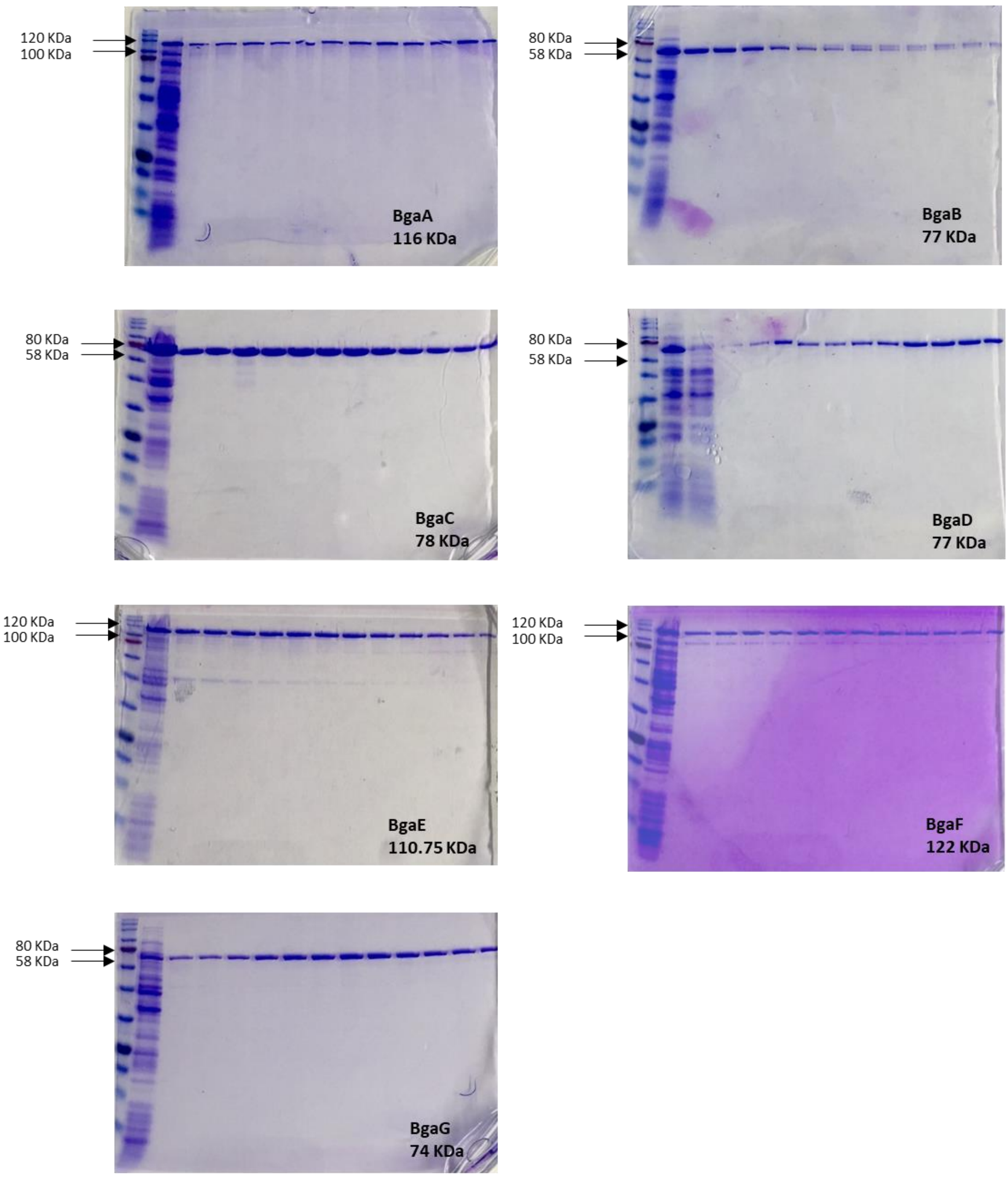

Figure S1. SDS-PAGE of purified proteins. Purified bifidobacterial β-galactosidases visualised by SDS-polyacrylamide gel electrophoreses (SDS-PAGE). The expected protein size is indicated underneath each enzyme name, while black arrows highlight the molecular weights of relevant molecular markers.

Enzymes stored at -20°C

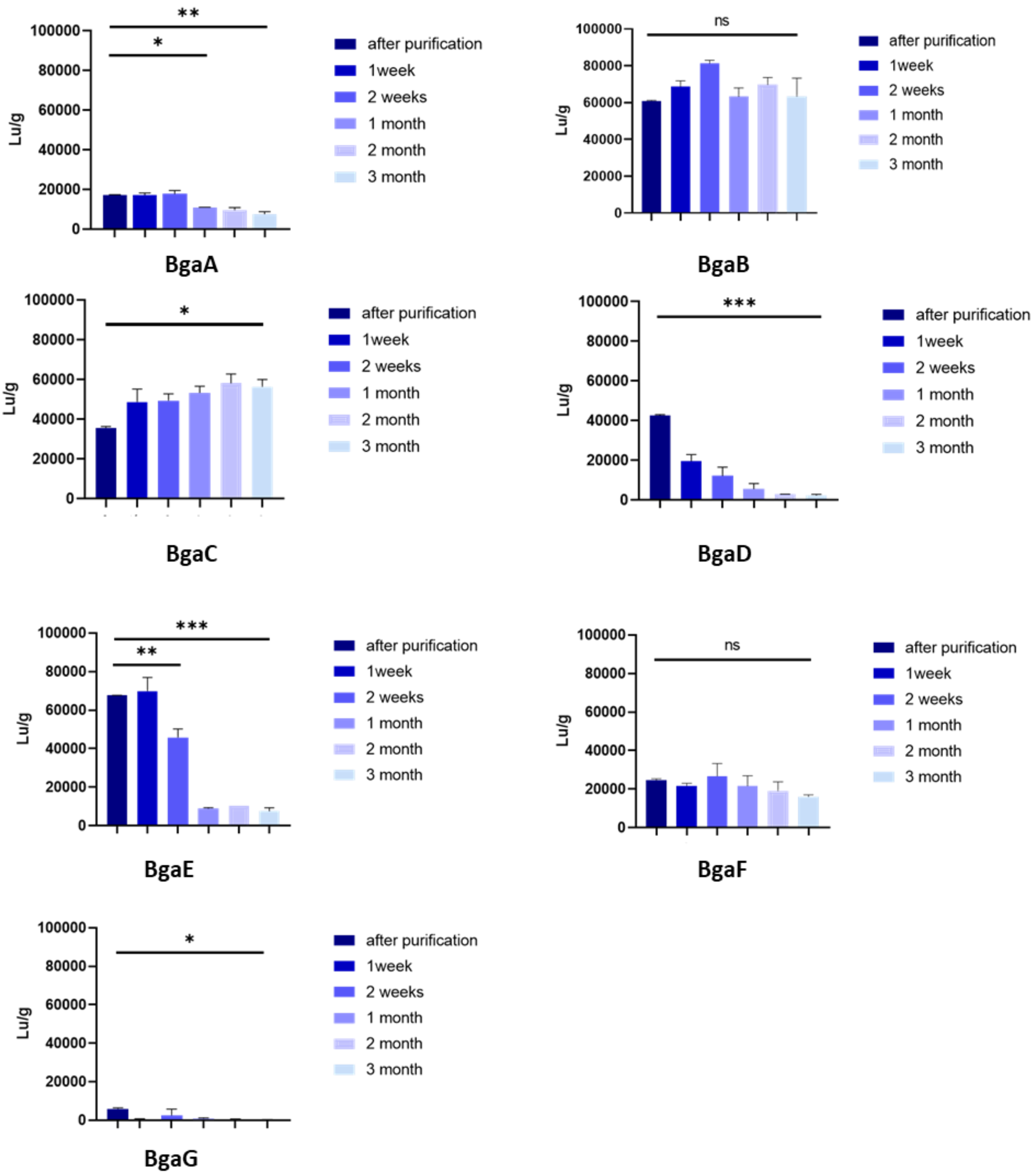

Figure S2. Enzyme stability assay at -20°C. Enzyme stability was measured over a period of 3 months. Lines represent the standard deviation of the mean (n=2). The asterisks indicate statistically significant differences:*: $p \leq 0.05$; **: $p \leq 0.001$; ***: $p \leq 0.0001$.

Enzymes stored at -20°C with glycerol

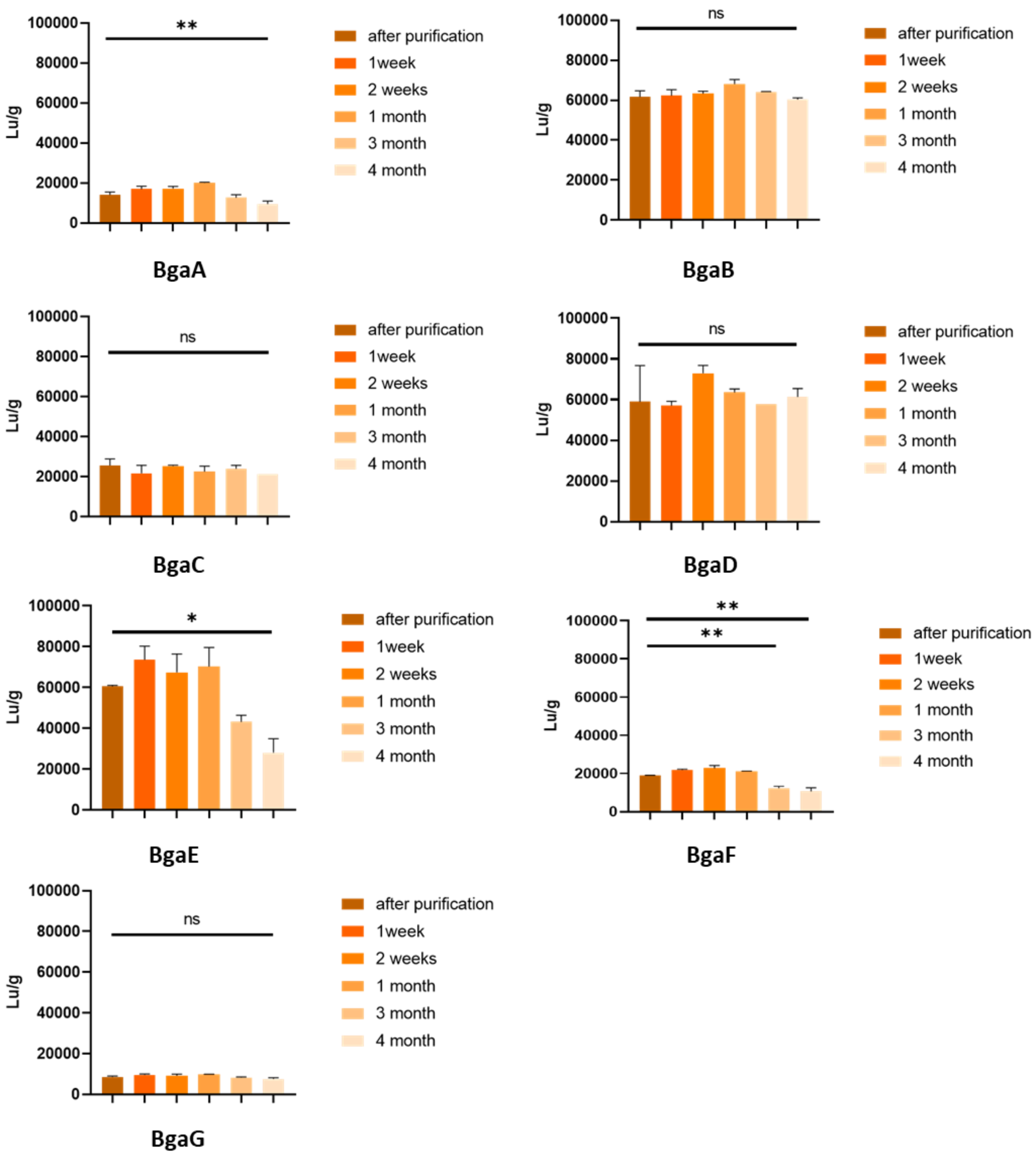

Figure S3. Enzyme stability assay at -20°C with glycerol. Enzyme stability was measured over a period of 4 months. Lines represent the standard deviation of the mean (n=2). The asterisks indicate statistically significant differences:*: $p \leq 0.05$; **: $p \leq 0.001$; ***: $p \leq 0.0001$.
